# Dynamic Response of Long Rectangular Floors Subjected to Periodic Force Excitation

**DOI:** 10.3390/ma12091417

**Published:** 2019-04-30

**Authors:** Zsuzsa B. Pap, László P. Kollár

**Affiliations:** Department of Structural Engineering, Faculty of Civil Engineering, Budapest University of Technology and Economics, Műegyetem rkp. 3., 1111 Budapest, Hungary; lkollar@eik.bme.hu

**Keywords:** long rectangular plates, dynamic amplification factor, periodic force excitation

## Abstract

Since damping in lightweight floors is usually low, dynamic amplification can be rather high. Long rectangular plates subjected to concentrated loads are often investigated by a replacement beam with a so called “effective width”. Although this approach is a reliable tool for static loads, the steady-state dynamic response of beams and long plates subjected to periodic loads are significantly different. The maximum displacements and accelerations of beams (and of not-long rectangular plates) are obtained by using a dynamic amplification factor, which in the case of resonance is equal to 1/2ξ, where ξ is the damping ratio. For long plates (and for not-long orthotropic rib-stiffened plates), as discussed in the paper, the response and the amplification factor are substantially different from those of beams. Hence, design based on effective width may lead to 2–4 times higher acceleration than the real values. In an economic design, to avoid unnecessary damping enhancement, this effect must be taken into account.

## 1. Introduction

Vibration control is a major consideration in the design of light-weight floors [1]. The response (acceleration or speed) of structures subjected to human- or machine-induced vibration is compared to the tolerance limit of human comfort [2,3,4,5]. Several recent publications deal with the analysis and design of rectangular plates for both static and dynamic loads [6,7,8,9].

In the analysis of floors, both the transient solution (high-frequency systems, impulse loads) and the steady-state solution (low-frequency systems, periodic loads) may play an important role [10]. In this paper, only the steady-state response is investigated for periodic force excitation. Both numerical and experimental observations show that the response is typically dominated by a single mode, and the analysis can often be simplified to a single degree of freedom (1DOF) system [11]. Determining the corresponding “modal mass”, the OS-RMS90 value [12,13], or the (rms) acceleration [10] can be calculated.

**Figure 1:**
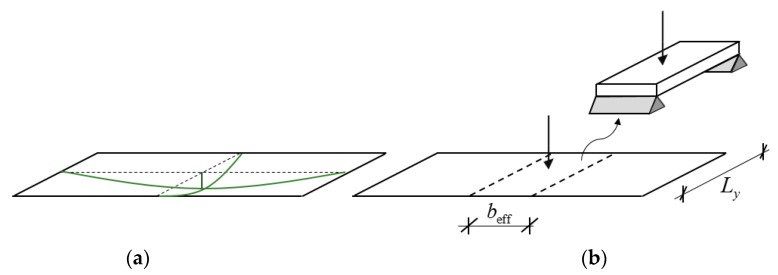
First eigen mode of a long plate (**a**) and the concept of effective width (**b**).

The above concept is sometimes applied for rectangular plates, which are long in one direction [14,15]. However, the longer the plate the higher the modal mass is; thus, for long plates the calculated response becomes unrealistically low. To overcome this problem, only an effective portion of the long plate is considered (Figure 1). A beam with a so-called “effective width” and with the corresponding modal mass is investigated instead of the long plate [16]. Maximum displacement and acceleration are obtained by using the modal mass and amplification factors, the latter ones depend on the damping ratio and on the ratio of the exciting- and eigen frequencies. Since damping in floors is usually low, this amplification can be rather high.

The effective width and corresponding modal mass are determined in such a way that the response of the 1DOF system is similar to that of the floor. For example, for the interior part of steel beam composite floors, based on measurements, the following expression is recommended [4,17]: $b_{\mathrm{eff}}=2\sqrt[{4}]{D_{11}/D_{22}}L_{y}$, with the upper limit of $b_{\mathrm{eff}}\leq 2/3L_{x}$, where $L_{y}$ is the span in the joist (*y*) direction, $L_{x}$ is the span in the *x* direction, while $D_{11}$ and $D_{22}$ are the bending stiffnesses for unit width in the *x* and *y* directions, respectively. If one of the harmonics of the load is in resonance with the floor, the response acceleration is calculated as a=(Q/M)/2ξ, where *Q* is the concentrated force amplitude in the corresponding Fourier term, *M* is the modal mass, and ξ is the damping ratio. Although this approach in a certain parameter range is rather accurate and easy to apply, it has an important shortcoming: in theory (Q/M) should be instantaneous acceleration and 1/2ξ is the maximum dynamic amplification factor; in reality, as it will be discussed in the paper, the instantaneous acceleration is usually higher, while the amplification factor is lower.

In other words, long plates (and not long orthotropic rib-stiffened plates) behave in a significantly different manner than beams or simple 1DOF mass–spring–damper systems. As a consequence, designs based on effective width may lead to 2–4 times higher accelerations than the real values. In an economic design, to avoid unnecessary damping enhancement, this effect must be taken into account.

## 2. Problem Statement and Approach

**Figure 2:**
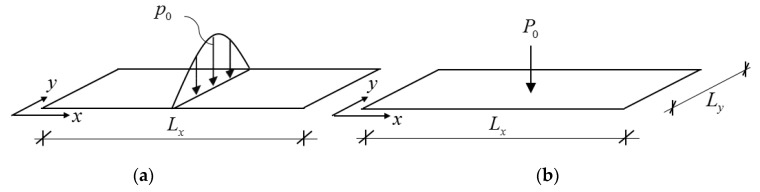
Long plate subjected to a trigonometrical line load (**a**) and to a concentrated load (**b**).

Rectangular orthotropic plates were considered, which were significantly longer in the *x* direction than perpendicular to it. (For orthotropic plates [15] $L_{x}>>\sqrt[{4}]{D_{11}/D_{22}}L_{y}$.) The edges parallel to the *x* coordinate were simply supported. Damping of the structure was characterized by the damping ratio *ξ*. The plate was subjected to either a sinusoidal line load or to a concentrated load (Figure 2). The load was periodic, and it was represented in time by its Fourier series expansion [18]. We wished to determine the steady-state response of the floor, the maximum displacement and acceleration, and investigate the applicability of the concept of effective width.

Orthotropic floors usually consist of parallel beams at least in one direction, and deflection of the plate between the beams may slightly influence the response. This effect will not be considered in the following analysis.

To solve the above problem for the trigonometrical load an analytical solution was applied, while for the concentrated load the FE analysis was applied.

Sinusoidal load can be considered as a one-term Fourier series expansion of the concentrated load ($p_{0}\to 2P_{0}/L_{y}$), and hence the responses from the two loads were expected to be similar. Although the sinusoidal load was not realistic, it had the important advantage that, for this case, an analytical solution could be derived.

## 3. Analytical Solution—Plates Subjected to Sine Line Loads

**Figure 3:**
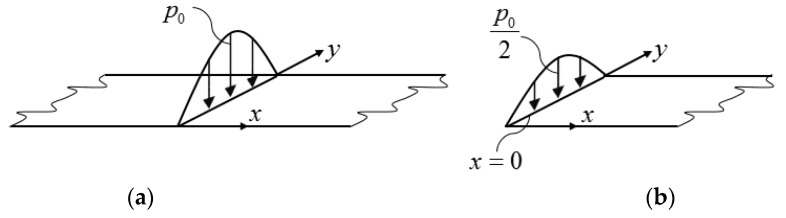
Long plate subjected to a trigonometrical line load (**a**) and the corresponding half plate (**b**).

An orthotropic plate was considered, where the governing partial differential equation was given by:

(1) $$\underset{\mathrm{bending}\;\;\mathrm{in}\;x\;\mathrm{direction}}{\underset{︸}{D_{11}\frac{\partial^{4}\tilde{w}}{\partial x^{4}}}}+\underset{\mathrm{torsion}}{\underset{︸}{2D_{t}\frac{\partial^{4}\tilde{w}}{\partial x^{2}\partial y^{2}}}}\;+\;\underset{\mathrm{bending}\;\;\mathrm{in}\;y\;\mathrm{direction}}{\underset{︸}{D_{22}\frac{\partial^{4}\tilde{w}}{\partial y^{4}}}}\;+\;m\frac{\partial^{2}\tilde{w}}{\partial t^{2}}=\tilde{p}\;,$$

where $\tilde{w}$ is the displacement perpendicular to the mid-plane, $D_{11}$ and $D_{22}$ are the bending stiffnesses, $D_{t}$ is the torsional stiffness, m is the mass per unit area, and $\tilde{p}$ is the distributed load of the plate. For isotropic plates and for Huber orthotropy [16] the torsional stiffness was $D_{t}=\sqrt{D_{11}D_{22}}$. For rib-stiffened plates the torsional stiffness was smaller than this value, in many cases significantly smaller. The middle line of the plate (*x* = 0) was loaded by a line load $p_{0}\sin(\pi y/L_{y})\sin(\omega t)$ (Figure 3a), or half of the plate was loaded by the line load (Figure 3b):

(2) $$\frac{p_{0}}{2}\sin\left(\frac{\pi y}{L_{y}}\right)\sin\left(\omega t\right)\;,$$

where the rest of the plate was unloaded. For the half plate there was a symmetry condition at *x* = 0 (i.e., the slope in the *x* direction was zero).

### 3.1. Levy’s Solution and Analogy with Beams on Elastic Foundation

Since the load varied with sine in the *y* direction, and the two edges parallel to the *x* axis were simply supported, following Levy’s approach [16] the displacement was assumed to be in the form of:

(3) $$\tilde{w}(x,y,t)=w(x,t)\sin\left(\frac{\pi y}{L_{y}}\right).$$

Introducing Equation (3) into Equation (1) we obtained the following ordinary differential equation:

(4) $$D_{11}\frac{\partial^{4}w}{\partial x^{4}}-2D_{t}\frac{\pi^{2}}{L_{y}^{2}}\frac{\partial^{2}w}{\partial x^{2}}+D_{22}\frac{\pi^{4}}{L_{y}^{4}}w+m\frac{\partial^{2}w}{\partial t^{2}}=p\;,$$

or

(5) $$D_{11}\frac{\partial^{4}w}{\partial x^{4}}-\vartheta \frac{\partial^{2}w}{\partial x^{2}}+\kappa w+m\frac{\partial^{2}w}{\partial t^{2}}=p\;,\;\vartheta =2D_{t}\frac{\pi^{2}}{L_{y}^{2}},\;\kappa =D_{22}\frac{\pi^{4}}{L_{y}^{4}}\;.$$

**Figure 4:**
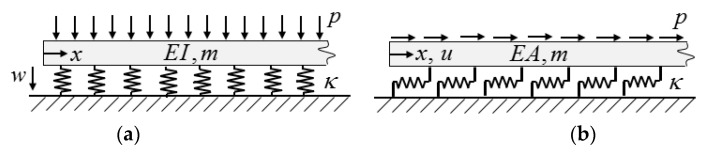
Beam on an elastic foundation (**a**) and a bar where the axial displacement is hindered by an elastic support (**b**).

Since there was no distributed load on the plate, *p* was zero. Note that when ϑ was set equal to zero, Equation (5) was identical to the equation of a beam on elastic foundation:

(6) $$EI\frac{\partial^{4}w}{\partial x^{4}}+\kappa w+m\frac{\partial^{2}w}{\partial t^{2}}=p\;,$$

where EI is the bending stiffness, *w* is the vertical displacement, and *κ* is the coefficient of the foundation (Figure 4a). Also note that when $D_{11}$ was set equal to zero, Equation (5) was identical to the equation of an axially loaded bar, the axial displacement of which was hindered by an elastic foundation:

(7) $$-EA\frac{\partial^{2}u}{\partial x^{2}}+\kappa u+m\frac{\partial^{2}u}{\partial t^{2}}=p\;,$$

where *EA* is the tensile stiffness, *u* is the *axial* displacement, *κ* is the coefficient of the foundation, and *p* is the axial load (Figure 4b). When beams or bars were infinitely long they behaved in a significantly different way than finite structures. There was a so-called cut-off frequency, calculated as [19]:

(8) $$\omega_{c}=\sqrt{\frac{\kappa }{m}}\;.$$

When the undamped beam or bar was loaded by a trigonometrical edge load (Equation (2)) at *x* = 0, for $\omega <\omega_{c}$ the steady-state solution showed no energy dissipation, and the displacements vanished far from the edge, while for $\omega >\omega_{c}$ there was energy dissipation (due to so-called “radiation damping”), and the displacement propagated far from the edge [19]. Since both models (Figure 4, Equations (6) and (7)) showed this behaviour, the complex structure (see Equation (5)) also behaved this way.

### 3.2. Effective Width and Dynamic Amplification Factor

First, the *static problem* was solved (sin(ωt) is replaced by unity in Equation (2)). Displacement in Equation (5) was assumed to be in the form of $w=e^{-\bar{\lambda }x}$, which, when introduced into the homogeneous form of Equation (5), gave the following characteristic equation:

(9) $$D_{11}{\bar{\lambda }}^{4}-\vartheta {\bar{\lambda }}^{2}+\kappa =0\;.$$

Assuming an infinitely long plate, and assuming symmetry boundary conditions at *x* = 0 (zero slope in the *x* direction), after a lengthy but straightforward algebraic manipulation, we obtained at *x* = 0:

(10) $$w_{\mathrm{stat}}=p_{0}A\;,\;A=\frac{1}{2\sqrt{\kappa \vartheta +2\sqrt{\kappa^{3}D_{11}}}}\;,$$

while the displacements vanished far from *x* = 0. 

**Figure 5:**
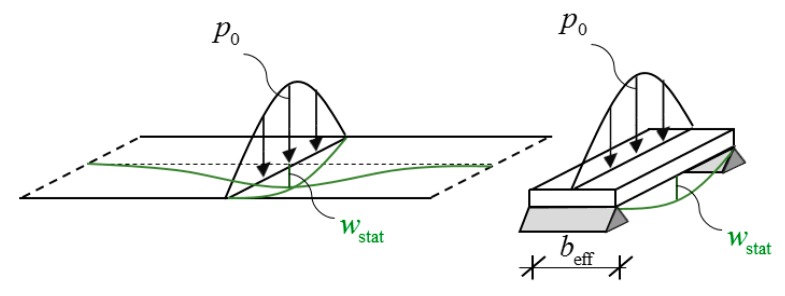
Methodology to determine the effective width.

Now an *effective width* was determined from the condition [16] that the maximum deflections of the plate at *x* = 0 and that of a beam with width $b_{\mathrm{eff}}$ for the same total load were identical (Figure 5):

(11) $$w_{\mathrm{stat}}=p_{0}A=p_{0}\frac{L_{y}^{4}}{\pi^{4}D_{22}b_{\mathrm{eff}}}\;=p_{0}\frac{1}{\kappa b_{\mathrm{eff}}}.$$

Equations (10), (11), and (5) give:

(12) $$b_{\mathrm{eff}}=2L_{y}\sqrt{\frac{\vartheta }{\kappa L_{y}^{2}}+2\sqrt{\frac{D_{11}}{\kappa L_{y}^{4}}}}=2\frac{L_{y}}{\pi }\sqrt{2\frac{D_{t}}{D_{22}}+2\sqrt{\frac{D_{11}}{D_{22}}}}\;.$$

This equation for isotropic plates $\left(D_{11}=D_{22}=D_{t}\right)$ simplified to: $b_{\mathrm{eff}}=(4/\pi )L_{y}=1.27L_{y}$, while for Huber orthotropy $\left(D_{t}=\sqrt{D_{11}D_{22}}\right)$: $b_{\mathrm{eff}}=(4/\pi )\sqrt[{4}]{D_{11}/D_{22}}L_{y}$. When there was no torsional stiffness $\left(D_{t}=0\right)$: $b_{\mathrm{eff}}=(2\sqrt{2}/\pi )\sqrt[{4}]{D_{11}/D_{22}}L_{y}=0.90\sqrt[{4}]{D_{11}/D_{22}}L_{y}$.

**Figure 6:**
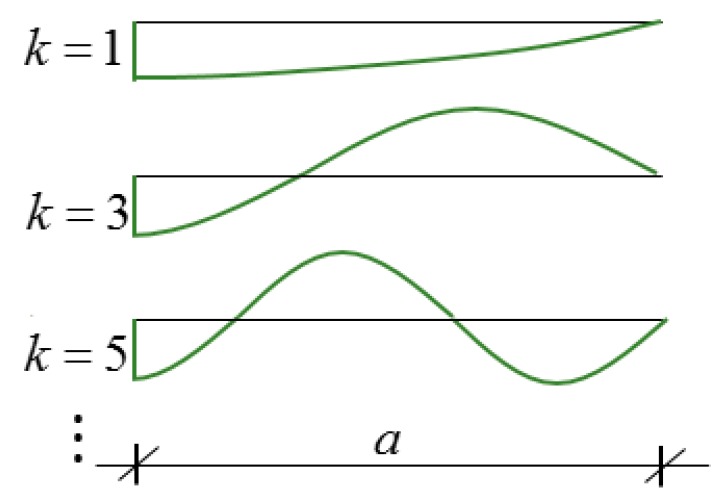
The displacement functions in Equation (13).

*Harmonic analysis*. Note that the undamped infinitely long system had an infinite number of eigenmodes and eigenfrequencies. For example, function (Figure 6):

(13) $$w(x,t)=\cos\left(\frac{k\pi x}{2a}\right)\;\sin\left(\omega t\right),$$

where any choice of *k* and a satisfies the symmetry condition at *x* = 0 and also the homogeneous differential equation (Equation (5)) when $\omega =\omega_{k}$. Here

(14) $$\omega_{k}=\omega_{c}\sqrt{1+{\left(\frac{k\pi }{2}\right)}^{2}\frac{\vartheta }{\kappa a^{2}}+{\left(\frac{k\pi }{2}\right)}^{4}\frac{D_{11}}{\kappa a^{4}}},$$

which is an eigenfrequency ($\omega_{c}$ is given by Equation (8)). Here we chose k=1,3,5… and a was set equal to such a high value that the solutions presented below were not sensitive to the variation of a. The load $p_{0}/2\sin(\omega t)$ at the edge was replaced by its Fourier series expansion as:

(15) $$p=\sum_{k=1,3,5\ldots }^{\infty }p_{k}\cos\left(\frac{k\pi x}{2a}\right)\sin\left(\omega t\right)\;,\;p_{k}=\frac{p_{0}}{a}\;,$$

where each term contained a *distributed* load along the plate. 

The displacement function was assumed to be in the form of the following series:

(16) $$w=\sum_{k=1,3,5\ldots }^{\infty }w_{k}\cos\left(\frac{k\pi x}{2a}\right)\sin\left(\omega t-\phi_{k}\right)\;,$$

where $w_{k}$ and $\phi_{k}$ are the yet unknown coefficients and phase angles. Introducing Equations (15) and (16) into Equation (5), each term could be solved separately, and the classical solution of the one degree of freedom systems was obtained for the undamped system [20]:

(17) $$w_{k}=\frac{p_{k}}{m\omega_{k}^{2}}D_{\delta k}\;,\;D_{\delta k}=\frac{1}{1-\beta_{k}^{2}}\;,\;\phi_{k}=0\;,\;\beta_{k}=\frac{\omega }{\omega_{k}}\;.$$

The *damped system* is investigated below, where damping was represented by the damping ratio ξ. Again, the classical solution of the one degree of freedom systems could be directly used [20]. Thus we had:

(18) $$w_{k}=\frac{p_{k}}{m\omega_{k}^{2}}D_{\delta k}\;,\;D_{\delta k}=\frac{1}{\sqrt{{\left(1-\beta_{k}^{2}\right)}^{2}-{\left(2\xi \beta_{k}\right)}^{2}}}\;,\;\phi_{k}=\tan^{-1}\left(\frac{2\xi \beta_{k}}{1-\beta_{k}}\right)\;,\;\beta_{k}=\frac{\omega }{\omega_{k}}\;.$$

The displacement at *x* = 0 could be given as (Equations (15), (16), and (18)):

(19) $$w=\sum_{k=1,3,5\ldots }^{\infty }w_{k}\sin\left(\omega t-\phi_{k}\right)\;=\sum_{k=1,3,5\ldots }^{\infty }\frac{p_{0}}{a}\frac{D_{\delta k}}{m\omega_{k}^{2}}\sin\left(\omega t-\phi_{k}\right)\;.$$

Equations (10), (14), (18), and (19) yield:

(20) $$w=p_{0}A\sum_{k=1,3,5\ldots }^{\infty }\frac{2\sqrt{\kappa \vartheta +2\sqrt{\kappa^{3}D_{11}}}}{\sqrt{1+{\left(\frac{k\pi }{2}\right)}^{2}\frac{\vartheta }{\kappa a^{2}}+{\left(\frac{k\pi }{2}\right)}^{4}\frac{D_{11}}{\kappa a^{4}}}}\frac{\sin\left(\omega t-\phi_{k}\right)}{\sqrt{{\left(1-\beta_{k}^{2}\right)}^{2}-{\left(2\xi \beta_{k}\right)}^{2}}}\;.$$

where *w* is a function of *t*. Its maximum value divided by the static displacement was the dynamic displacement amplification factor:

(21) $$D_{d}=\frac{\max\left[w\left(t\right)\right]}{w_{\mathrm{stat}}}=\frac{\max\left[w\left(t\right)\right]}{p_{0}A}\;,$$

and from the *t* value where the maximum occurred ($t_{\max}$), we obtained the phase angle of the system:

(22) $$\phi =\omega t_{\max}-\pi /2\;.$$

The steady state acceleration and the dynamic acceleration amplification factors were:

(23) $$a=w_{\mathrm{stat}}\omega^{2}D_{d}=w_{\mathrm{stat}}\omega_{c}^{2}\frac{\omega^{2}}{\omega_{c}^{2}}D_{d}=\frac{p_{0}}{mb_{\mathrm{eff}}}D_{a}\;,\;D_{a}\;=\frac{\omega^{2}}{\omega_{c}^{2}}D_{d}\;.$$

**Figure 7:**
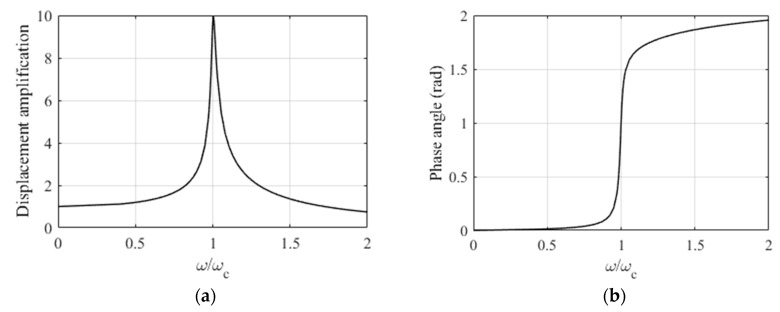
Displacement amplification factor (**a**) and phase angle (**b**) of an infinitely long isotropic plate subjected to a trigonometrical line load.

*D*_d_ and ϕ can be calculated from Equations (20)–(22). An example for an isotropic plate with ξ=1% is given in Figure 7. For a finite system the value of the amplification factor at the resonance point was:

(24) $$D_{\delta r}=\frac{1}{2\xi }={\left(2\xi \right)}^{-1}\;,\;$$

for ξ=1% it was $D_{\delta r}=50$. Note that in this example, because the plate was infinitely long, the maximum value was significantly smaller: $D_{d,r}=9.94$. (This result was also verified by harmonic analysis in ANSYS 14.5 Mechanical APDL software.)

Numerically we determined the peak values for several cases, and a curve was fitted for the interval 1%≤ξ≤5%. It was found that for a beam on an elastic foundation (ϑ=0, Equation (6)) the amplification factor at resonance was:

(25) $$D_{d,r}^{0}\approx \frac{1}{{\left(2\xi \right)}^{0.75}}\;,\;$$

while for a bar on an elastic foundation ($D_{11}=0$, Equation (7)):

(26) $$D_{d,r}\approx \frac{1}{\sqrt{2\xi }}.$$

For a plate without torsional stiffness and for an isotropic (or Huber orthotropic) plate, the amplification factors at resonance could be approximated as:

(27) $$D_{d,r}^{0}\approx \frac{1}{{\left(2\xi \right)}^{0.75}},\;D_{d,r}^{H}\approx \frac{1}{{\left(2\xi \right)}^{0.6}}.$$

The dynamic responses of a long plate at resonance were (Equations (11) and (27)):

(28) $$w_{\mathrm{dyn}}^{0}\approx \underset{w_{\mathrm{stat}}}{\underset{︸}{\frac{p_{0}L_{y}^{3}}{\pi^{4}D_{22}0.90}\sqrt[{4}]{\frac{D_{22}}{D_{11}}}}}\frac{1}{{\left(2\xi \right)}^{0.75}}\;,\;w_{\mathrm{dyn}}^{H}\approx \underset{w_{\mathrm{stat}}}{\underset{︸}{\frac{p_{0}L_{y}^{3}}{\pi^{4}D_{22}1.27}\sqrt[{4}]{\frac{D_{22}}{D_{11}}}}}\frac{1}{{\left(2\xi \right)}^{0.6}}\;,$$

where the first expression was valid for a plate without torsional stiffness, and the second expression was valid for a plate with Huber orthotropy (and for an isotropic plate). To obtain the maximum accelerations, the above expressions must be multiplied by $\omega^{2}=\omega_{c}^{2}=D_{22}\pi^{4}/mL_{y}^{4}$:

(29) $$a_{\mathrm{dyn}}^{0}\approx \frac{p_{0}}{\underset{b_{\mathrm{eff}}}{\underset{︸}{0.90\sqrt[{4}]{D_{11}/D_{22}}L_{y}}}m}\frac{1}{{\left(2\xi \right)}^{0.75}}\;,\;a_{\mathrm{dyn}}^{H}\approx \frac{p_{0}}{\underset{b_{\mathrm{eff}}}{\underset{︸}{1.27\sqrt[{4}]{D_{11}/D_{22}}L_{y}}}m}\frac{1}{{\left(2\xi \right)}^{0.6}}\;.$$

### 3.3. Plates with Finite Length

**Figure 8:**
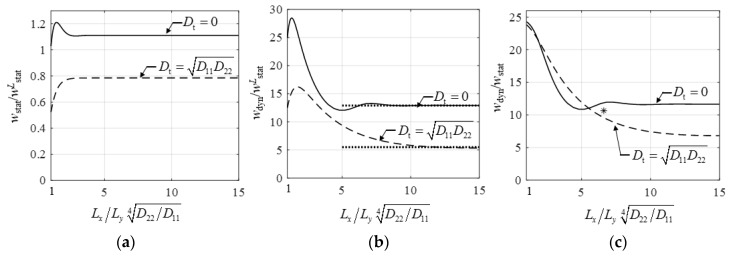
(**a**) Static displacement, (**b**) dynamic displacement of the steady-state solution of a plate excited by $\sin(\omega_{c}t)$, and (**c**) dynamic amplification factor as a function of the plate aspect ratio (the displacements are normalized by the static displacement of an *L*_y_ wide plate strip: $w_{\mathrm{stat}}^{L}=p_{0}L_{y}^{3}/\pi^{4}D_{22}$). Equation (28) is given in (**b**) by dotted lines. The result of the numerical example is shown by an asterisk in (**c**).

Infinitely long plates were considered above. Now it is investigated how the amplification factor and the phase angle depend on the aspect ratio of the plate. In Figure 8 the static and the dynamic responses are given as a function of the plate length. Two extreme cases of torsional stiffness were considered, on the lower curves $D_{t}=\sqrt{D_{11}D_{22}}$, while on the upper curves $D_{t}=0$. As expected, the static displacement slightly changed if the aspect ratio was above 3 ($L_{x}\sqrt[{4}]{D_{22}/D_{11}}/L_{y}>3$). The dynamic response deteriorated only above aspect ratios of 6–10. Dynamic displacement had a maximum, about $L_{x}\sqrt[{4}]{D_{22}/D_{11}}/L_{y}=1.7$, for plates with torsional stiffness, and about $L_{x}\sqrt[{4}]{D_{22}/D_{11}}/L_{y}=1.3$ for plates without torsional stiffness. Equation (28) is given in Figure 8b by dotted lines.

**Figure 9:**
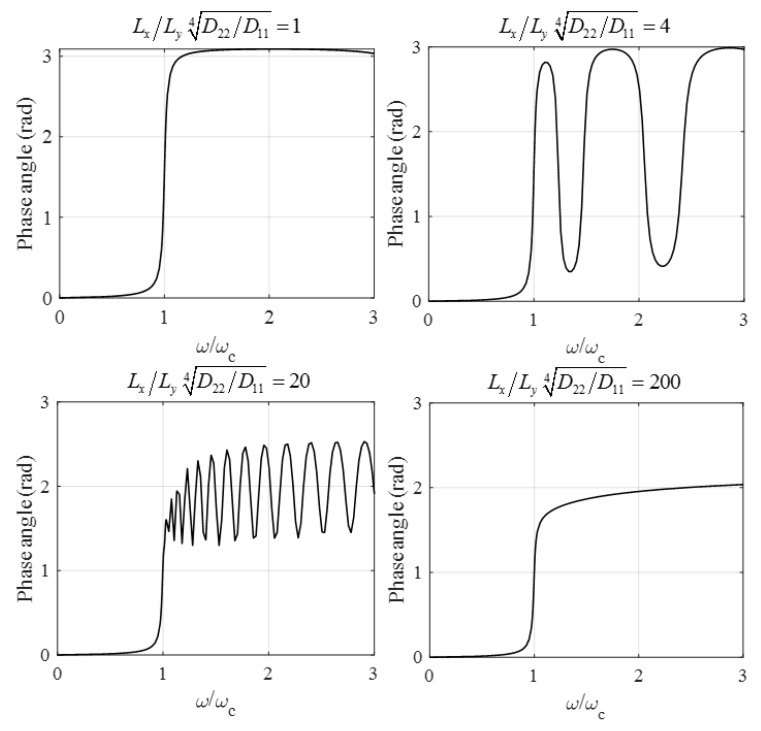
Phase angle of plates with finite length subjected to a trigonometrical line load $\left(\xi =1\%,\;\kappa =\pi^{4}\;N/m^{3},\;\vartheta =2\pi^{2}N/m,\;D_{11}/\kappa \;=1\;m^{4},\;\omega_{c}=1\;\;1/s\right)$.

The phase angle is shown in Figure 9 for $D_{t}=\sqrt{D_{11}D_{22}}$.

## 4. Numerical Solution—Plates Subjected to Concentrated Loads

**Figure 10:**
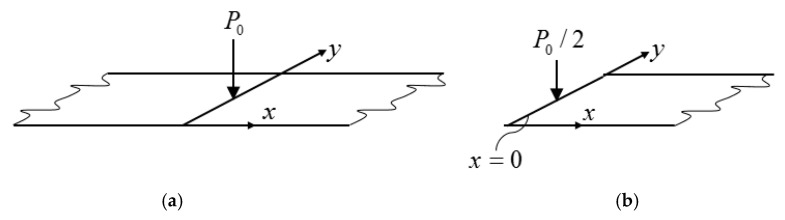
Long plate subjected to a concentrated load (**a**) and the corresponding half plate (**b**).

Now the plate was loaded by a concentrated load $P_{0}\sin(\omega t)$ at the middle (Figure 10a), or half of the plate was loaded by the concentrated load (Figure 10b):

(30) $$\frac{P_{0}}{2}\;\sin\left(\omega t\right)\;,$$

and the rest of the plate was unloaded. Analysis was carried out with the ANSYS 14.5 Mechanical APDL FE software. In the finite element model SHELL181 4-node elements, simply supported boundary conditions, and a linear, orthotropic material model were used. For the calculation of the infinitely long plate, the length of the plate was chosen high enough that the solution was not sensitive to the variation of length. At least 300 elements were used along the width of the plate, and the results were also verified by the benchmark solutions of the literature [16]. The aspect ratio of the elements was set to one. 

First, using a static analysis the maximum deflection and the effective width were determined from the condition that the maximum deflection of the plate at *x* = 0 and that of a beam with width $b_{\mathrm{eff}}$ for the same total load were identical:

(31) $$w_{\mathrm{stat}}=P_{0}\frac{L_{y}^{3}}{48D_{22}b_{\mathrm{eff}}}\;.$$

(Note that the one-term Fourier approximation of the concentrated load, $p_{0}\approx 2P_{0}/L_{y}$ gave the constant $\pi^{4}/2=48.7$ instead of 48.) For isotropic plates $\left(D_{11}=D_{22}=D_{t}\right)$ we obtained numerically: $b_{\mathrm{eff}}=1.23L_{y}$, while for Huber orthotropy $\left(D_{t}=\sqrt{D_{11}D_{22}}\right)$: $b_{\mathrm{eff}}=1.23\sqrt[{4}]{D_{11}/D_{22}}L_{y}$. These results were identical to the classical solution [16]. When there was no torsional stiffness $\left(D_{t}=0\right)$: $b_{\mathrm{eff}}=0.88\sqrt[{4}]{D_{11}/D_{22}}L_{y}$. These values were less than 4% from those given for sinusoidal loads. 

**Figure 11:**
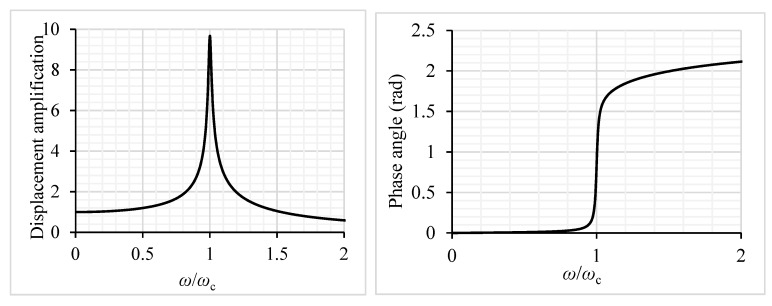
Displacement amplification factor and phase angle of an infinitely long isotropic plate subjected to a concentrated load (*D*_d,r_ = 9.67).

*Harmonic analysis*. The dynamic problem of the plate subjected to a concentrated load applying the harmonic analysis of the ANSYS software was solved, The results are shown in Figure 11. Note that the plate subjected to a line (sine) load was presented in Figure 7; the maximum difference was only 2.7%.

The dynamic response of a long plate at resonance can be approximated as (Equations (27) and (31)):

(32) $$w_{\mathrm{dyn}}^{0}\approx \underset{w_{\mathrm{stat}}}{\underset{︸}{\frac{P_{0}L_{y}^{2}}{48D_{22}0.88}\sqrt[{4}]{\frac{D_{22}}{D_{11}}}}}\frac{1}{{\left(2\xi \right)}^{0.75}}\;,\;w_{\mathrm{dyn}}^{H}\approx \underset{w_{\mathrm{stat}}}{\underset{︸}{\frac{P_{0}L_{y}^{2}}{48D_{22}1.23}\sqrt[{4}]{\frac{D_{22}}{D_{11}}}}}\frac{1}{{\left(2\xi \right)}^{0.6}}\;,$$

where the first expression is valid for a plate without torsional stiffness, while the second one for a plate with Huber orthotropy. To obtain the maximum accelerations, the above expressions must be multiplied by $\omega_{c}^{2}=D_{22}\pi^{4}/mL_{y}^{4}$:

(33) $$a_{\mathrm{dyn}}^{0}\approx \frac{P_{0}}{0.5\times \underset{b_{\mathrm{eff}}L_{y}}{\underset{︸}{0.88\sqrt[{4}]{D_{11}/D_{22}}L_{y}^{2}}}m}\frac{1}{{\left(2\xi \right)}^{0.75}}\;,\;a_{\mathrm{dyn}}^{H}\approx \frac{P_{0}}{0.5\times \underset{b_{\mathrm{eff}}L_{y}}{\underset{︸}{1.23\sqrt[{4}]{D_{11}/D_{22}}L_{y}^{2}}}m}\frac{1}{{\left(2\xi \right)}^{0.6}}\;.$$

## 5. Numerical Example

**Figure 12:**
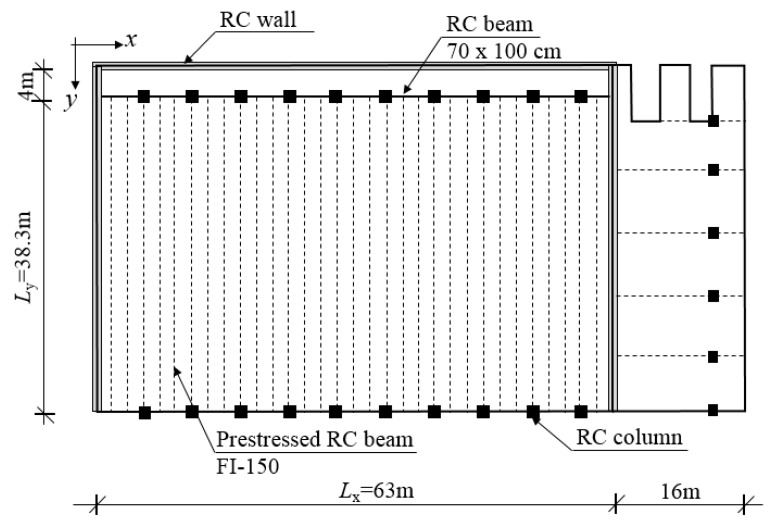
Geometry of the analyzed floor.

**Figure 13:**
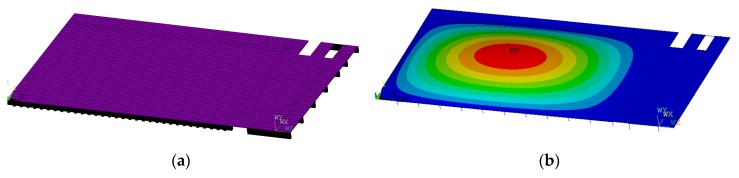
(**a**) FE model of the analyzed slab and (**b**) first eigen shape of the slab *f*_0_ = 1.97 Hz.

The roof of a swimming pool was examined, which was designed in Budapest and will be built in the coming year. The slab was made of prefabricated, prestressed RC beams of height 1.5 m and an RC slab with 200 mm thickness, which were connected by steel shear connectors. The sizes of the floor were $L_{x}=63\;m$ and $L_{y}=38.3\;m$, the mass of the floor was *m* = 1830 kg/m^2^, and the bending stiffnesses were $D_{11}=24.2\;\mathrm{MNm},\;D_{22}=6216\;\mathrm{MNm}$. The damping ratio was ξ=2%. The geometry of the slab is shown in Figure 12. The FE model of the floor was built in ANSYS. SHELL181 elements were used for the slab, BEAM188 elements for the beams, and MPC184 elements for the connection between the slab and beams (Figure 13a). The vibration calculation was performed in a load level when the materials were linear, therefore only the stiffnesses and density of the materials were set. The concrete was assumed to be uncracked for the calculation. The slab was supported in the vertical direction under the walls and columns, and it was supported by springs in *x* and *y* directions under the columns. The slab had been under design; experiments and measurements had not been performed yet.

**Figure 14:**
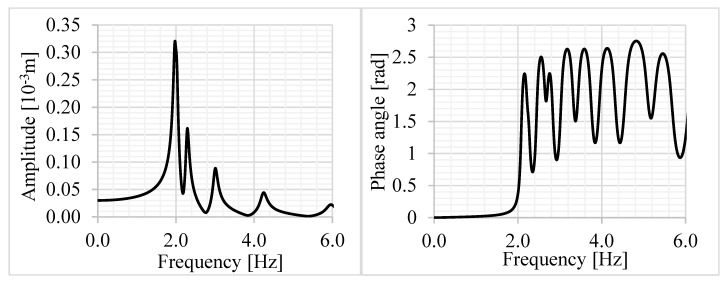
Dynamic response of the analyzed slab for a concentrated force excitation.

The first eigenfrequency of the floor was 1.97 Hz (Figure 13b), which was very close to the frequency of walking; this meant resonance would occur in case of human activity. The floor was investigated for the rhythmic activity of a group of people, here only harmonic analysis was presented for one concentrated harmonic force (*P*_0_ = 1.8 × 746 N, first harmonic of one jumping person) at the middle of the floor. The amplitude of vertical displacement and the phase angle is shown in Figure 14.

The dynamic amplification was *D*_d,r_ = 10.65 and the phase angle was 126° (Figure 14) at the resonance frequency. Although the aspect ratio of the floor was $L_{x}/L_{y}=1.64$, because of orthotropy we had $L_{x}\sqrt[{4}]{D_{22}/D_{11}}/L_{y}=6.61$. Dynamic displacement and acceleration were 0.32 mm and 1.28 mm/s^2^, respectively. Results of the numerical example are shown in Figure 8c by an asterisk. We observed that because of high anisotropy the floor could be considered infinitely long, and the dynamic amplification factor was significantly smaller than 1/2*ξ* = 25. When—as an approximation—torsional stiffness was neglected, Equations (32) and (33) resulted in:

(34) $$w_{\mathrm{dyn}}^{H}\approx \underset{w_{\mathrm{stat}}}{\underset{︸}{\frac{P_{0}L_{y}^{2}}{48D_{22}1.23}\sqrt[{4}]{\frac{D_{22}}{D_{11}}}}}\frac{1}{{\left(2\xi \right)}^{0.75}}=0.336\;\mathrm{mm}\;,$$

(35) $$a_{\mathrm{dyn}}^{H}\approx \frac{P_{0}}{0.5\times 1.23\sqrt[{4}]{D_{11}/D_{22}}L_{y}^{2}m}\frac{1}{{\left(2\xi \right)}^{0.75}}=1.308\frac{\mathrm{mm}}{s^{2}}\;.$$

which were close to the above numerical values.

## 6. Comparison of the Response of Long Plates to Single Degree of Freedom (1DOF) Systems

It was shown that the dynamic amplification factors at resonance of long plates (and long beams on an elastic foundation) were significantly smaller than 1/2ξ, which belonged to a 1DOF structure. The same statement was true for non-long rib-stiffened plates, which were highly orthotropic. The reason for this was that long plates had several closely spaced eigenvalues, and although the individual modes behaved analogously to a 1DOF structure, in the overall response those of several modes must be added together. Thus, the effect of damping was magnified. This might be explained also by the role of radiation damping. 

**Figure 15:**
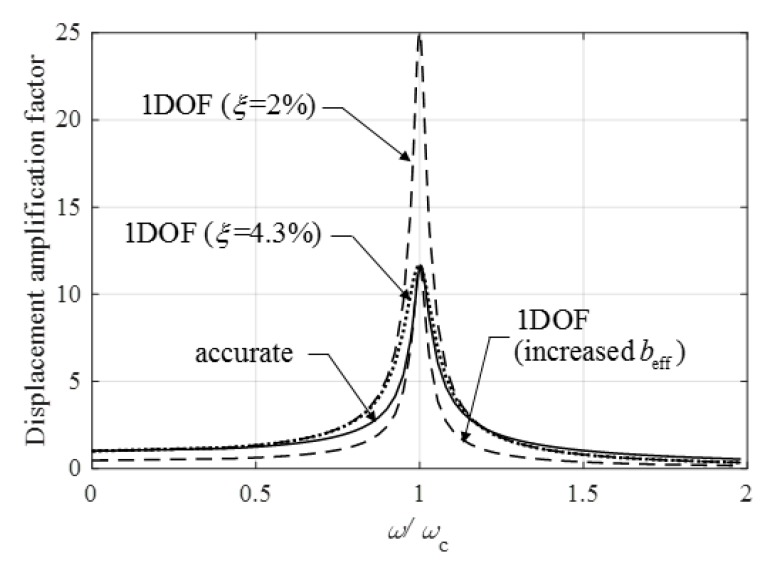
Displacement amplification factor of an infinitely long orthotropic plate (ξ=2%) without torsional stiffness subjected to a trigonometrical line load (solid line) and those of one degree of freedom (1DOF) systems. Dashed lines give the response calculated for two different effective widths (ξ=2%), while the dotted line is obtained from a replacement damping ratio $\xi_{\mathrm{repl}}=4.3\%$.

In Figure 15 the dynamic amplification factor of an infinitely long orthotropic plate, without torsional stiffness, subjected to a trigonometrical line load, is given by a solid line and compared to the response of a 1DOF system, which belonged to a beam with the effective width given by Equation (12) (see the upper dashed line). In both models the damping ratio was ξ=2%. The two lines were close to each other for low-exciting frequency, but there were huge differences close to resonance. 

A possible remedy for the difference at the peak value was that that the effective width was increased in such a way that the dynamic response of a 1DOF system (using 1/2ξ) at resonance became identical to that of the floor. The corresponding curve was given by the lower dashed line in Figure 15. It could be observed that the differences were huge everywhere except close to resonance. (In this case, dynamic displacement was normalized by accurate static displacement.)

It seems a reasonable approximation that the effective width was calculated for static response (Equation (11)), but the damping was increased (because of ”radiation damping”) to match the peak values:

(36) $$\xi_{\mathrm{repl}}=\frac{1}{2D_{d.r}}.$$

Since for the investigated case $D_{d,r}=11.6$, the replacement damping ratio was $\xi_{\mathrm{repl}}=1/\left(2\times 11.6\right)=4.3\%.$ The corresponding curve of the 1DOF was given by a dotted line (Figure 15). Note that although the peak value and the static response were accurate, the response curve of the 1DOF structure differed from that of the floor. 

## 7. Comments on the Current Design Recommendations

Let us investigate now the concept of replacement beams with an effective width given in [15] and in the design recommendation [17]. The effective width (for long plates) is given by the expression $b_{\mathrm{eff}}^{C}=2\sqrt[{4}]{D_{11}/D_{22}}L_{y}$ [17], which results in the following displacement and acceleration at resonance:

(37) $$w_{\mathrm{dyn}}^{C}=\frac{P_{0}L_{y}^{2}}{48D_{22}\times 2}\sqrt[{4}]{\frac{D_{22}}{D_{11}}}\frac{1}{2\xi },\;a_{\mathrm{dyn}}^{C}=\frac{P_{0}}{0.5\times 2\sqrt[{4}]{D_{11}/D_{22}}L_{y}^{2}m}\frac{1}{2\xi }.$$

**Figure 16:**
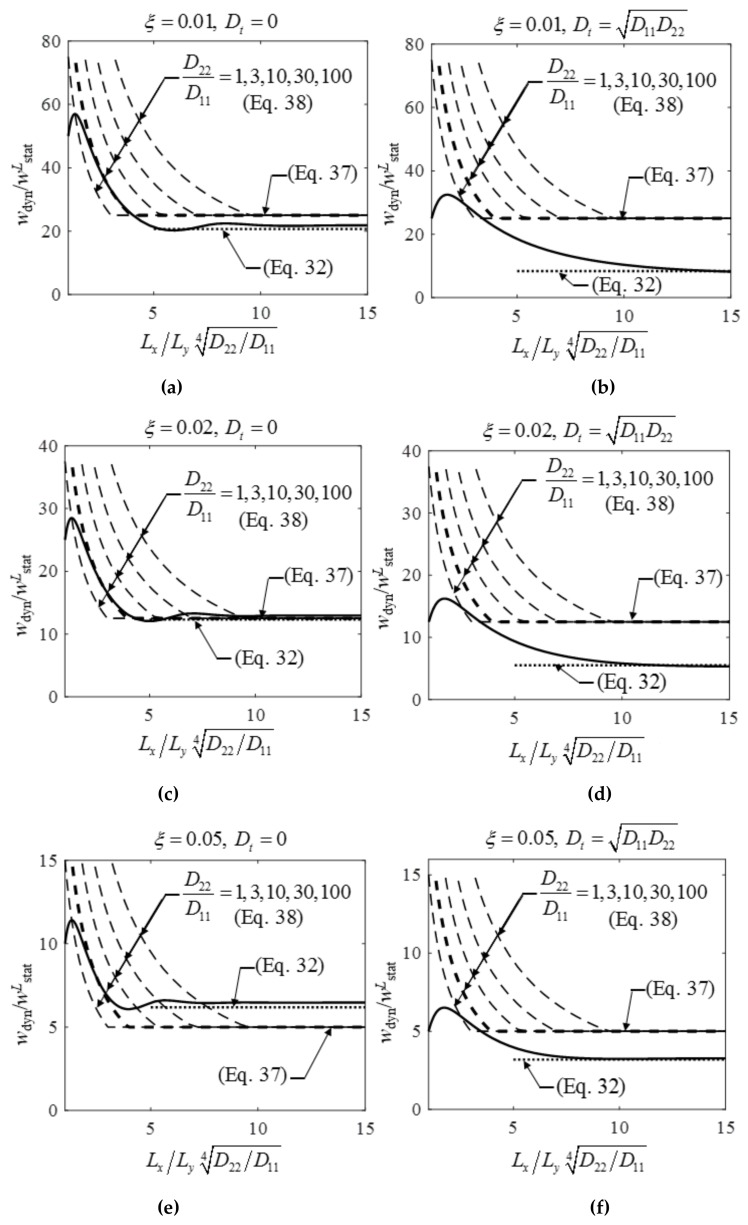
Dynamic displacements of a plate subjected to a concentrated load at resonance. The displacements are normalized by the static response of an *L*_y_ wide plate strip $w_{\mathrm{stat}}^{L}=P_{0}L_{y}^{2}/48D_{22}$. On the left the plate has no torsional stiffness while on the right $D_{t}=\sqrt{D_{11}D_{22}}$. The three rows correspond to *ξ* = 1%, 2%, and 5% damping ratios. The accurate solution is given by black continuous lines, Equations (37) and (38) by dashed lines, and Equation (32) by dotted lines.

In Figure 16 the “accurate” plate displacements at resonance are given by solid lines, while Equation (37) by dashed *horizontal* lines. For no torsional stiffness, which was a reasonable approximation for steel beam composite floors, the results for high aspect ratios were reasonable; for ξ=2% they were practically accurate. (Note that this replacement width was valid only for the dynamic response at resonance, see the lower dashed line in Figure 15). Our results (Equation (28)) were also given in Figure 16 by dotted lines, which were very accurate for high aspect ratios. 

For shorter floors, according to [17] the limit value of the effective width must be applied: $b_{\mathrm{eff}}^{C}\leq 2/3L_{x}$. By using this expression, we obtain:

(38) $$w_{\mathrm{dyn}}^{C}=\frac{P_{0}L_{y}^{3}}{48D_{22}\times 2/3L_{x}}\frac{1}{2\xi }.$$

The corresponding curves in Figure 16 depended on $D_{22}/D_{11}$. Five curves were given for $D_{22}/D_{11}=1,\;3,\;10,\;30,\;100$. (The value of a steel beam composite floor was about 30.) It was clear that for realistic stiffness values this approximation could be very inaccurate. 

If instead of the above limit

(39) $$b_{\mathrm{eff}}^{C}\leq 1/2\sqrt[{4}]{D_{22}/D_{11}}L_{x}$$

is applied, we obtain one curve

(40) $$w_{\mathrm{dyn}}^{C}=\frac{P_{0}L_{y}^{3}}{48D_{22}\times 1/2L_{x}}\sqrt[{4}]{\frac{D_{22}}{D_{11}}}\frac{1}{2\xi }$$

for an arbitrary stiffness ratio, as shown in Figure 16, by fat dashed lines, which are close to the solid line, when there is no torsional stiffness and $L_{x}>1.5\sqrt[{4}]{D_{11}/D_{22}}L_{y}$.

To summarize the results of our calculations, the recommendation of [17] for long orthotropic plates without torsional stiffness (at resonance) was acceptable, however, the limit value of the effective width for shorter floors should be modified as given in the previous paragraph.

Although impulse load was not the task of this paper, it is important to mention that approximation of replacement widths, where for shorter plates the dynamic response is independent of the stiffness ratio, might be highly inaccurate.

## 8. Conclusions

The major finding of our paper is that rectangular orthotropic floors with realistic dimensions may behave in a significantly different way than 1DOF structures, and their maximum amplification factor is much lower than 1/2ξ (Figure 8c). In an economic design, to avoid unnecessary damping enhancement this effect must be taken into account. To prove this, an analytical derivation is performed for a sine line load, and FE analysis is made for a concentrated load. The main steps of the derivation are presented by Equations (10)–(29). Our suggestion is that the amplification factor (for long plates without torsional stiffness and for long isotropic plates) can be calculated according to Equation (27) instead of 1/2ξ. The maximum displacement and acceleration are given by Equations (28) and (29) for sine line loads and by Equations (32) and (33) for concentrated loads.

Both sinusoidal and concentrated loads were investigated. It was found that the corresponding effective widths, amplification factors, and phase angles were close to each other, and the effect of damping was magnified, which can be explained by the role of radiation damping. The effect of enhanced damping and, thus, the reduction in the dynamic amplification factor must be taken into account in the design of floors.

It was shown that the results based on the effective width defined by the design specification [17], and based purely on experiments, can be obtained also numerically by the model of an orthotropic plate without torsional stiffness. Since this agreement also validates our model, and the limit given in the specification (Equation (38)) could not be verified, further research should be made on this expression. Numerically we found that Equation (40) can be used (as shown in Figure 16 by fat dashed lines); however, this should also be verified experimentally. 

## References

[1] Bachmann H., Ammann W. (1987). Vibrations in Structures Induced by Man and Machines.

[2] (2018). ISO 10137:2007 (E) Bases for Design of Structures—Serviceability of Buildings and Walkways against Vibrations. https://www.iso.org/obp/ui/#iso:std:iso:10137:ed-2:v1:en.

[3] Allen D.E., Rainer J.H. (1976). Vibration criteria for long-span floors. Can. J. Civ. Eng..

[4] Allen D.E., Murray T.M. (1992). Design criterion for walking vibrations. ASCE J. Struct. Eng..

[5] Allen D.E., Rainer J.H., Pernica G. (1985). Vibration criteria for assembly occupancies. Can. J. Civ. Eng..

[6] Xing Y., Sun Q., Liu B., Wang Z. (2018). The overall assessment of closed-form solution methods for free vibrations of rectangular thin plates. Int. J. Mech. Sci..

[7] Liu C., Ke L.L., Yang J., Kitipornchai S., Wang Y.S. (2018). Nonlinear vibration of piezoelectric nanoplates using nonlocal Mindlin plate theory. Mech. Adv. Mater. Struc..

[8] Zhao J., Wang Q., Deng X., Choe K., Zhong R., Shuai C. (2019). Free vibrations of functionally graded porous rectangular plate with uniform elastic boundary conditions. Compos. Part B Eng..

[9] Haciyev V.C., Sofiyev A.H., Kuruoglu N. (2018). Free bending vibration analysis of thin bidirectionally exponentially graded orthotropic rectangular plates resting on two-parameter elastic foundations. Compos. Struct..

[10] Smith A.L., Hicks S.J., Devine P.J. (2007). Design of Floors for Vibration: A New Approach.

[11] Hicks S. (2004). Vibration characteristics of steel–concrete composite floor systems. Prog. Struct. Eng. Mat..

[12] Feldmann M., Heinemeyer C., Völling B. (1996). Design Guide for Floor Vibrations.

[13] Feldmann M., Heinemeyer C., Butz C., Caetano E., Cunha A., Galanti F., Goldack A., Hechler O., Hicks S.J., Keil A. (2009). Design of Floor Structures for Human Induced Vibrations. https://www.google.com.tw/url?sa=t&rct=j&q=&esrc=s&source=web&cd=1&ved=2ahUKEwiuqfPB0fbhAhV2yIsBHe-VAzAQFjAAegQIBRAC&url=https%3A%2F%2Feurocodes.jrc.ec.europa.eu%2Fshow_Entity.php%3Ffile_id%3DEC_00000069&usg=AOvVaw28czFequ5OVAT0uPwO7E07.

[14] Mateus B., Dietrich Z., Teixeira F.B., Fernanda A., Calenzani G., Ferreira W.G. (2014). Vibrations in Steel-Frame Floors Due to Human Activities. https://globaljournals.org/GJRE_Volume14/1-Vibrations-in-Steel-Frame.pdf.

[15] Allen D.E., Murray T.M. (1993). Design criterion for vibrations due to walking. Eng. J..

[16] Timoshenko S., Woinowsky-Krieger S. (1959). Theory of Plates and Shells.

[17] Murray T.M., Allen D.E., Ungar E.E., Davis D.B. (2016). Vibrations of Steel-Framed Structural Systems Due to Human Activity.

[18] Rao S.S. (2007). Vibration of Continuous Systems.

[19] Hagedorn P., DasGupta A. (2007). Vibrations and Waves in Continuous Mechanical Systems.

[20] Clough R.W., Penzien J. (2003). Dynamics of Structures.

